# 2015 update of the evidence base: World Allergy Organization anaphylaxis guidelines

**DOI:** 10.1186/s40413-015-0080-1

**Published:** 2015-10-28

**Authors:** F. Estelle R. Simons, Motohiro Ebisawa, Mario Sanchez-Borges, Bernard Y. Thong, Margitta Worm, Luciana Kase Tanno, Richard F. Lockey, Yehia M. El-Gamal, Simon GA Brown, Hae-Sim Park, Aziz Sheikh

**Affiliations:** Department of Pediatrics & Child Health and Department of Immunology, College of Medicine, Faculty of Health Sciences, The University of Manitoba, Room FE125, 820 Sherbrook Street, Winnipeg, R3A 1R9 MB Canada; Department of Allergy, Clinical Research Center for Allergy & Rheumatology, Sagamihara National Hospital, Sagamihara, Kanagawa Japan; Allergy and Clinical Immunology Department, Centro Medico-Docente La Trinidad, Caracas, Venezuela; Department of Rheumatology, Allergy & Immunology, Tan Tock Seng Hospital, Singapore, Singapore; Allergie-Centrum-Charite, Klinik fur Dermatologie, Venerologie und Allergologie, Campus Charite Mitte, Universitatsmedizin, Berlin, Germany; Department of Allergy and Clinical Immunology, Hospital Servidor Publico Estadual de Sao Paulo and Hospital Sirio-Libanes, Sao Paulo, Brazil; University of South Florida College of Medicine, Tampa, FL USA; Pediatric Allergy and Immunology Unit, Children’s Hospital, Ain Shams University, Cairo, Egypt; Royal Hobart Hospital, Tasmania, and University of Western Australia and Royal Perth Hospital, Perth, Western Australia; Department of Allergy & Clinical Immunology, Ajou University School of Medicine, Seoul, South Korea; Allergy & Respiratory Research Group, Usher Institute of Population Health Sciences and Informatics, The University of Edinburgh, Edinburgh, UK

**Keywords:** Anaphylaxis, Epinephrine, Auto-injector, Food allergy, Stinging insect venom allergy, Drug allergy, Latex allergy, Exercise-induced anaphylaxis, Systemic allergic reaction, Adrenaline

## Abstract

The World Allergy Organization (WAO) Guidelines for the assessment and management of anaphylaxis provide a unique global perspective on this increasingly common, potentially life-threatening disease. Recommendations made in the original WAO Anaphylaxis Guidelines remain clinically valid and relevant, and are a widely accessed and frequently cited resource. In this 2015 update of the evidence supporting recommendations in the Guidelines, new information based on anaphylaxis publications from January 2014 through mid- 2015 is summarized. Advances in epidemiology, diagnosis, and management in healthcare and community settings are highlighted. Additionally, new information about patient factors that increase the risk of severe and/or fatal anaphylaxis and patient co-factors that amplify anaphylactic episodes is presented and new information about anaphylaxis triggers and confirmation of triggers to facilitate specific trigger avoidance and immunomodulation is reviewed. The update includes tables summarizing important advances in anaphylaxis research.

## Introduction

The World Allergy Organization (WAO) Guidelines for the Assessment and Management of Anaphylaxis remain a clinically relevant, widely accessed, and frequently cited resource [1]. This 2015 Update is intended for use in conjunction with these WAO Anaphylaxis Guidelines, the 2012 and 2013 Updates of the evidence supporting the Guidelines, and the 2014 International Consensus on (ICON): Anaphylaxis, a publication with a broader mandate than the Updates [2–4].

Here, we review research published in peer-reviewed indexed journals from January 2014 through mid-2015 on the epidemiology of anaphylaxis, patient risk factors, amplifying co-factors, triggers, diagnosis, and management in healthcare and community settings. We also highlight excellence in clinical and translational research relevant to anaphylaxis and cite major publications from other allergy/immunology professional organizations [5, 6] and different global regions.

### Epidemiology

Updated information about the epidemiology of anaphylaxis, a life-threatening systemic or hypersensitivity reaction with sudden onset, comes from general population surveys and secondary analyses of large clinical and administrative databases [7–16] (Table 1).

**Table 1:** **Epidemiology and patient risk factors**

**Epidemiology [** 7 **–** 16 **]**
Hospitalization rates for anaphylaxis continue to increase year-on-year [9–12].
Anaphylaxis fatality rates have remained stable or decreased slightly. Fatalities are age-, co-morbidity-, and trigger-related [12–14].
The highest hospital admission rates for food-induced anaphylaxis occur in very young children age 0–4 years; however, the rate of increase in the age groups 5–14 years and 15–29 years is accelerating [11].
**Patient risk factors and amplifying co-factors [** 17 **–** 37 **]**
Data from an anaphylaxis registry of more than 5000 patients with systemic allergic reactions indicated that although monotherapy with beta-blockers and to a lesser extent, ACE inhibitors, increased the risk of severe anaphylaxis, the risk was further increased by concurrent use of a drug from each class [26].
In an experimental model, although a beta-blocker (metoprolol) given alone had a modestly aggravating effect and an ACE inhibitor (ramipril) given alone had no significant effect, anaphylaxis was exacerbated and mediator release was increased when these medications were given concurrently [26].
*In vitro*, FcεRI-mediated mast cell histamine release was not increased by metoprolol or ramipril given alone, but was synergistically increased when the drugs were given together. This three-part study provides epidemiologic and experimental evidence that ACE inhibitors and beta-blockers aggravate anaphylaxis partly as a result of direct mast cell priming and decreasing the threshold for mast cell activation [26].
In a prospective study of co-factors that amplify anaphylaxis, wheat-dependent, exercise-induced anaphylaxis occurred when plasma gliadin levels were elevated by ingestion of higher gluten doses, gluten and exercise, or gluten and acetylsalicylic acid plus alcohol [36].

*ACE* angiotensin-converting enzyme inhibitor

The lifetime risk of symptoms suggestive of anaphylaxis in the general population, as reported by members of the public, is at least 1.6 %. This estimate is based on a survey of 1000 unselected US adults with a sudden-onset illness involving two or more body organ systems, including the respiratory and/or cardiovascular systems, who sensed their lives were in danger and received hospital care [8].

The frequency of hospital admissions for anaphylaxis is increasing. Information obtained from the US Healthcare Cost and Utilization Project Kids’ In-Patient Database consisting of a stratified random sample of 12,039,432 hospital discharges indicated that admissions for food-induced anaphylaxis in children aged <18 years more than doubled from 2000 to 2009, with corresponding increases in associated healthcare costs [9]. Similarly, analysis of the Italian Ministry of Health database revealed that hospitalizations for food-induced anaphylaxis in children increased year-on-year from 2006 through 2011. The increase was more pronounced in those aged 5–14 years than in those age 4 years or younger [10]. Time trends in Australian hospitalization rates for food-induced anaphylaxis indicate that admissions continue to increase across all age groups. Although the highest hospitalization rates occurred in children age 0–4 years, the greatest *acceleration* in the rates of increase was found in the age groups 5–14 years and 15–29 years [11].

In England and Wales, hospital admissions from all-cause anaphylaxis increased by 615 % from 1992 to 2012; however, fatalities, checked against a prospective fatal anaphylaxis registry, remained stable at 0.047 cases/million population. Admission rates and fatality rates for anaphylaxis from drugs and insect stings were highest in the elderly. Admission rates for food-induced anaphylaxis were highest in young people, with a sharp peak in the incidence of food anaphylaxis fatalities in the second and third decades of life [12].

From 1999 to 2009, although hospitalizations for anaphylaxis increased in the USA (annual percentage change 2.2 %), this contrasted with a decreased case fatality rate among emergency department (ED) patients and hospitalized patients (annual percentage change −2.35 %). Overall mortality rates ranged from 0.63 - 0.76/million population (186–225 deaths per year) and appeared stable during the decade studied [13]. In another review of 2458 anaphylaxis fatalities from 1999 to 2010, drugs (58.8 %) were the most common trigger followed by unspecified inducers (19.3 %), venoms (15.2 %) and foods (6.7 %). Fatalities were associated with older age and other demographic factors [14].

Anaphylaxis has been misclassified in both the International Classification of Diseases (ICD)-9 and ICD-10 versions. The global allergy community views the 11th revision, ICD-11, as an opportunity to improve the classification and coding of hypersensitivity/allergic diseases and is aiming to have a specific chapter on these diseases included to facilitate future epidemiological studies [15, 16].

### Patient risk factors and amplifying co-factors in anaphylaxis

Many publications now include information on patient risk factors and amplifying co-factors in anaphylaxis [17–37] (Table 1). These risk factors and co-factors differ from one age group to another. They are not yet optimally studied in the pediatric population.

In infancy, there is a paucity of anaphylaxis data because of under-recognition and under-diagnosis due to age-unique symptom patterns. In order to increase awareness of anaphylaxis in this age group, illustrated pathways for clinical diagnosis, management and prevention of recurrences in infants have been developed [19], based on the WAO Guidelines [1].

In teenagers, there is an increased risk of severe and/or fatal anaphylaxis, as reported in several different types of studies. For example, in those who developed life-threatening anaphylaxis during food oral immunotherapy (OIT) studies, relevant endogenous risk factors included being an adolescent and having uncontrolled asthma, while relevant exogenous co-factors included lack of compliance with asthma preventer medications and/or with OIT protocols, in addition to exercise, fasting, denial of symptoms, and delay in seeking help [20].

In pregnancy, anaphylaxis is uncommon, but potentially catastrophic because it can place mothers and infants at high risk for hypoxic-ischemic encephalopathy or death. Performing skin tests and challenge tests and initiating allergen immunotherapy are typically avoided because of the associated small, although definite, risk of anaphylaxis. A new algorithm for the investigation of anaphylaxis to RhD immunoglobulin G (anti-D) has been proposed to minimize the need for anti-D challenges in pregnant Rh-negative women. It includes a detailed history, limited skin testing, and fetal Rh genotyping from maternal blood [1, 2, 21].

Old age, combined with co-morbidities such as cardiovascular disease (CVD) and chronic obstructive pulmonary disease, is an important risk factor for severe anaphylaxis with hospitalization, prolonged hospital stay, and fatality [12, 14, 17, 18, 22, 23]. Some widely used life-saving medications for CVD such as beta-blockers and angiotensin-converting enzyme (ACE) inhibitors appear to increase the risk of, or severity of, anaphylaxis; however, their interactions and mechanisms of action are still incompletely understood [23–25].

In a registry involving more than 5000 patients with acute allergic reactions, there was a higher risk of severe anaphylaxis in patients taking a beta-blocker and an ACE inhibitor concurrently than when a beta-blocker or an ACE inhibitor were given alone [26]. These findings were confirmed in an experimental model in which metoprolol given alone had some aggravating effect and ramipril given alone had no significant effect, but anaphylaxis symptoms were exacerbated and mediator release was increased when they were given in combination [26]. *In vitro*, FcεRI-mediated mast cell histamine release was increased by metoprolol and ramipril given together, whereas these substances had no significant effect when given alone; the mechanism likely involved direct mast cell priming and a decrease in the threshold for mast cell activation [26].

Systemic mastocytosis may predispose to severe life-threatening anaphylaxis. Although many patients with this disorder have few or no mediator-related symptoms, others have recurrent episodes of severe anaphylaxis with clear signs of a mast cell activation syndrome (MCAS). Some patients with systemic mastocytosis have IgE-dependent symptoms; however, the severity and frequency of MCAS reactions do not correlate with specific IgE levels, basal tryptase levels, or the burden of neoplastic mast cells [27]. Other patients have unexplained recurrent episodes of severe anaphylaxis with CVD symptoms such as syncope and elevated basal tryptase levels (>11.4 mcg/L) [28].

Patients who have indolent systemic mastocytosis (ISM) *without* skin signs, and anaphylaxis triggered exclusively by insect stings, have clinical and laboratory features that differ significantly from other patients with ISM. In this distinct subgroup, males predominate, basal tryptase levels are not greatly elevated, *KIT* mutations are frequently restricted to bone marrow mast cells, and there are few bone marrow mast cell aggregates [29].

A rapid inexpensive screening test for the *KIT* D816V mutation in peripheral blood has been developed. This test facilitated recognition of systemic mastocytosis in 4/113 consecutive adult patients with anaphylaxis who had normal or low basal serum tryptase levels and absent or inconspicuous urticaria pigmentosa skin lesions [30].

More studies on elevation of basal levels of mediators as a risk factor for anaphylaxis are being published. Elevated tryptase levels are found to be a good marker for predicting *Hymenoptera* venom-induced anaphylaxis [31]. In a controlled study, low basal levels of platelet-activating factor (PAF) acetylhydrolase (leading to increased PAF levels) were also associated with severe venom anaphylaxis [32]. A large extended family with Mendelian inheritance of elevated basal tryptase levels included members with MCAS, anaphylaxis, atopy, and connective tissue abnormalities [33].

Co-factors, including exercise, ethanol, non-steroidal anti-inflammatory drugs (NSAIDs), acute infections, stress, and perimenstrual status potentially amplify anaphylaxis by decreasing the threshold of allergen exposure (the allergen “dose”) needed to trigger anaphylaxis and by amplifying the risk of anaphylaxis in patients with low or borderline allergen sensitization [20, 34–37].

The proposed pathophysiologic mechanisms underlying EIA require prospective testing [35]. As an example, in a controlled study in 16 adults with a history of wheat-dependent, exercise-induced anaphylaxis (WDEIA) and omega-5-gliadin-specific IgE, prospective oral food challenges (OFCs) with increasing amounts of gluten alone, or in combination with one or more co-factors, were performed until symptoms developed. Plasma gliadin levels were elevated by higher gluten doses, gluten and exercise, or gluten and acetylsalicylic acid (ASA) plus alcohol. Positive plasma gliadin threshold levels differed by more than 100-fold (median 628 pg/mL, range 15–2111). In some patients, exercise was not an essential trigger for symptoms [36].

Perimenstrual anaphylactic episodes in girls and women are attributed to various mechanisms such as hypersensitivity to progesterone or prostaglandin. Estrogen might also play a role by enhancing endothelial expression of nitric oxide synthase and nitric oxide production, increasing vascular permeability, and intensifying anaphylaxis severity [37].

### Triggers of anaphylaxis

Globally important triggers (inducers) of anaphylaxis such as foods, stinging insect venoms, and drugs remain a major research focus [38–77] (Table 2).

**Table 2:** **Food, insect sting, and drug/iatrogenic triggers**

*Food* [34, 38–49]
In a prospective study, 10/12 patients with IgE-mediated mammalian meat-induced systemic allergic reactions had OFCs with meat. Symptom onset occurred in 3–7 hours and correlated with basophil activation and increased CD63 expression, likely reflecting the appearance of galactose-alpha 1,3-galactose (alpha-gal) in the blood. Controls remained asymptomatic after OFCs [45].
Attributed to higher alpha-gal concentrations (or more accessible alpha-gal epitopes), pork kidney elicited symptoms and positive skin tests more consistently than pork muscle meat. Co-factors, including alcohol, NSAIDs, and exercise, amplified low levels of alpha-gal sensitization [34].
*Hymenoptera Venom* [50–54]
The relevance of asymptomatic sensitization to *Hymenoptera* venom was tested in 94 people who had previously tolerated field stings without a systemic allergic reaction. On screening, they had elevated venom-specific IgE levels. They underwent a complete evaluation (skin tests, component-resolved diagnosis, BATs, and physician-supervised insect sting challenges). Large local reactions occurred in 43.6 %, and systemic reactions occurred in 5.3 %. Current venom allergy tests did not distinguish asymptomatic sensitization from risk of local or systemic reactions [52].
The predictive value of *in vitro* tests with *Hymenoptera* venoms can be improved by use of recombinant venom allergens and component resolved testing, for example, by utilizing a panel of honeybee allergens that are free from cross-reactive carbohydrate determinants [53, 54].
*Drugs/Iatrogenic Agents* [55–77]
The health consequences of penicillin “allergy” included costly longer admissions, greater use of broad-spectrum antibiotics, and increased prevalence of serious infections with *Clostridium difficile*, methicillin-resistant *Staphylococcus aureus*, and vancomycin-resistant enterococci [58].
Risk factors for fatal anaphylaxis to a neuromuscular blocker included male sex, emergency setting, CVD/hypertension, concurrent beta-blocker treatment, and obesity [71].
Of 228 consecutive patients with perioperative anaphylaxis, 9.6 % were sensitized to the antiseptic/disinfectant chlorhexidine, as determined using specific IgE measurements, basophil activation tests, and standardized skin tests [73].

*alpha-gal* galactose-alpha 1,3-galactose; *BATs* basophil activation tests, *CVD* cardiovascular disease, *NSAIDs* non-steroidal anti-inflammatory drugs; *OFCs* oral food challenges

In a large European registry study, standardized data are being collected from patients with anaphylaxis referred to allergy/immunology clinics, facilitating accurate comparison of triggers, symptom patterns, and treatment trends across countries [38].

### Food-induced anaphylaxis

The immunopathology of food-induced anaphylaxis, tests to confirm specific triggers, and approaches to management have been reviewed in depth [39, 40]. Foods are by far the most common anaphylaxis trigger in infants, children, teens, and young adults [9–12]. As reported in a meta-analysis of data from 34 studies, the incidence rate of food-induced anaphylaxis was 0.14 per 100 person-years at all ages, and up to 7 per 100 person-years in children aged 0–4 years [41].

In a retrospective study of 168 adults aged >18 years with new-onset food allergy, symptoms typically began in the second and third decades; 49 % of these patients reported anaphylaxis. Shellfish, tree nuts, fish, soy, and peanut accounted for most cases, although many other foods were implicated [42].

A 3 year follow-up study in 80 children with ED visits for systemic allergic reactions (SARs) to foods who had 116 repeat ED visits confirmed that severity of previous reactions did not predict severity of subsequent reactions [43].

In the past two decades, cashew has become an important allergen due to increased consumption. Like other tree nuts and peanuts, it contains potent allergens that can trigger severe anaphylaxis when ingested in trace amounts. Cross-reactivity with pistachio is reported [39, 44].

In a prospective controlled study, 10 of 12 patients with a history of urticaria after ingestion of mammalian meat developed reactions ranging from urticaria to anaphylaxis within 3–7 hours of an open OFC with meat. Basophil activation (increased CD63 expression) correlated with symptom onset, likely reflecting antigen (galactose-alpha 1,3-galactose [alpha-gal]) appearance in the blood. None of the 13 control subjects developed symptoms [45].

In patients with alpha-gal-induced anaphylaxis, pork kidney elicited symptoms and positive skin prick tests (SPT) more consistently than pork muscle meat, attributed to higher alpha-gal concentrations (or more accessible alpha-gal epitopes) in pork kidney compared with muscle. Co-factors, including alcohol, NSAIDs, and exercise amplified low levels of sensitization to alpha-gal [34].

Anaphylaxis has been reported from Japan in patients who ingested okonomiyaki pancakes made from mite-contaminated pancake mix and who had elevated specific IgE to *Dermatophagoides* and *Tyrophagus* species [46].

In pharmaceutical formulations and other manufactured products, foods and food derivatives used as excipients include: egg protein, egg lecithin, fish protamine, fish oil, gelatin (bovine or porcine), casein, lactalbumin, lactose, peanut oil, sesame oil, soy lecithin, soy oil, and soy phosphatidylcholine [47]. Anaphylaxis has been reported in children with milk allergy and asthma treated with lactose-containing intravenous (IV) formulation of methylprednisolone sodium succinate [48], and life-threatening anaphylaxis has been reported after topical exposure to casein in an Everlast kickboxing glove [49].

### Insect sting or bite-induced anaphylaxis

Many people in the general population who have tolerated previous insect stings are sensitized to *Hymenoptera* venom [50, 51]. In a prospective study to test the relevance of asymptomatic sensitization, 94 such individuals had venom skin tests (prick and intradermal), specific IgE levels, component-resolved diagnosis, and basophil activation tests (BATs), followed by physician-supervised challenge stings from one or more live insects. Large local reactions occurred in 43.6 % (a 9.5-fold increased risk *versus* asymptomatic, unsensitized individuals), and SARs occurred in 5.3 %, suggesting that currently available tests cannot distinguish asymptomatic sensitization from the risk of large local reactions or SARs [52].

Patients who are clinically allergic to *Hymenoptera* venom yet have absent or undetectable venom-specific IgE can be identified by measuring serum IgE to a panel of recombinant honeybee and yellow jacket venom allergens (rVes v 1, rVes v 2, rVes v 3, and rVes v 5, and rApi m 1, rApi m 2, rApi m 3 and rApi m 5). Use of recombinant allergens significantly increases test sensitivity, as compared with specific IgE measurements to commercial venom extracts [53].

Improved *in vitro* tests for patients with honeybee venom allergy are being developed. Analysis of a panel of cross-reactive carbohydrate determinant-free honeybee venom allergens improved diagnostic sensitivity compared with use of rApi m 1 alone, identified additional major allergens, and revealed sensitization to allergens that have been reported to be absent or under-represented in commercial venom extracts [54].

### Drug-induced anaphylaxis

Drugs, most commonly antibiotics, NSAIDs, and neuromuscular blocking agents (NMBAs), are frequent triggers of anaphylaxis. Drug-induced anaphylaxis is under-recognized. It can also be over-diagnosed based on history alone. Allergy/immunology evaluations are under-utilized. Immediate reactions occurring within one hour after dosing are typically mediated by specific IgE. Skin testing is validated for beta-lactam antibiotics, but less so for other antibiotics and for other drug classes. Drug provocation tests are useful in selected patients [55–57].

A history of penicillin “allergy” has important health consequences. In a cohort of 51,582 hospitalized US patients *versus* matched controls, those with a history of penicillin “allergy” had significantly longer admissions, greater use of broad-spectrum antibiotics including fluoroquinolones, clindamycin, and vancomycin, and increased prevalence of serious infections including *Clostridium difficile*, methicillin-resistant *Staphylococcus aureus*, and vancomycin-resistant enterococcal infections [58]. In a UK teaching hospital, the total cost of antibiotics prescribed for patients reporting “penicillin allergy” was 1.8 to 2.6-fold higher than for first-line antibiotics [59].

Assessment by history and skin tests can rule out the likelihood of an acute allergic reaction to penicillins in 90-95 % of patients with a history of penicillin allergy, potentially leading to large cost savings in health systems. Skin testing with commercially available benzyl penicilloyl-polylysine (PrePen), previously validated in adults, has now also been validated in children [60].

Physician-documented cephalosporin anaphylaxis is rare. In a large US healthcare system, anaphylaxis occurred during only five oral cephalosporin exposures among 901,908 oral courses and during only eight parenteral cephalosporin exposures among 487,630 parenteral courses [61].

Although many penicillin-allergic patients can tolerate aztreonam or carbapenems, rare cross-reactivity between penicillins and these drugs has been reported. Of 212 consecutive patients with a penicillin allergy history and positive skin tests to one or more penicillins, all had negative skin tests to aztreonam and three carbapenems (imipenem-cilastatin, meropenem, and ertapenem). Of the 211 patients who consented to challenge, all passed. Pre-treatment skin tests with aztreonam and carbapenems are nevertheless still recommended because negative tests indicate tolerability [62].

In contrast, skin tests cannot be relied on to confirm hypersensitivity to quinolones. Drugs such as moxifloxacin frequently produce positive skin test responses in healthy individuals, and testing with high concentrations does not distinguish between patients with a history of reactions and control patients who have been exposed [63].

Few cases of anaphylaxis to macrolide antibiotics such as erythromycin or clarithromycin have been reported to date. Azithromycin-induced anaphylaxis has been confirmed by skin testing in three children [64].

NSAIDs are major triggers of anaphylaxis [65, 66]. In the Portuguese Pharmacovigilance system, over 4 years, they were responsible for 47.9 % of drug-induced anaphylaxis episodes and 25.6 % of anaphylaxis recurrences. Implicated drugs included preferential COX-1 inhibitors such as ASA, diclofenac, ibuprofen, naproxen, and, uncommonly, weak COX-1 inhibitors such as paracetamol (acetaminophen) [66].

Nine cases of IgE-dependent anaphylaxis to diclofenac were recorded over a decade by the Allergy Vigilance Network in France. Sensitization to diclofenac was confirmed in four of these patients by positive intradermal tests, in one patient who developed acute urticaria after intradermal testing, and in one skin test-negative patient who developed anaphylaxis after an oral challenge with diclofenac. The investigators emphasized the need for development of tests to measure specific IgE to frequently used NSAIDs such as diclofenac and ibuprofen [67].

Administration of any drug by any route can potentially cause anaphylaxis. As an example, 3.5 % of 230 patients developed anaphylaxis within one hour of subcutaneous injection of the plasma kallikrein inhibitor ecallantide. There was no consistent association with IgE to ecallantide or to *Pichia pastoris*, the yeast cells in which ecallantide is produced by recombinant DNA technology [68].

Hypersensitivity reactions ranging from mild skin reactions to life-threatening anaphylaxis from biological agents such as rituximab, trastuzumab, cetuximab, ofatumumab, tocilizumab, and brentuximab are increasing in frequency as use of these potentially life-saving agents increases in neoplastic, auto-immune and inflammatory diseases. Desensitization enables patients to receive a full treatment regimen of these agents without developing anaphylaxis [69].

Perioperative anaphylaxis, a serious complication, is reported in up to 1/13,000 anesthetics. It can be caused by NMBAs such as atracurium, suxamethonium, rocuronium, or vecuronium; or by antibiotics, blood and blood products, dyes, chlorhexidine, or natural rubber latex. In a prospective study, 13 of 4595 (1 in 353) patients having a general anesthetic met referral criteria for investigation of perioperative anaphylaxis, suggesting that the problem is under-diagnosed [70]. From 2000 to 2011, 2022 cases of NMBA anaphylaxis, 84 (4.1 %) of which were fatal, were reported to the French National Pharmacovigilance Network. Independent risk factors associated with death were: male gender, emergency setting, history of hypertension or other CVD, ongoing beta-blocker treatment, and obesity [71].

In 14 of 15 patients with perioperative anaphylaxis due to sugammadex injection for reversal of neuromuscular blockade, symptoms began within four minutes of the sugammadex injection [72].

Chlorhexidine, an antiseptic/disinfectant active against a broad spectrum of bacteria, viruses, and fungi, is an increasingly common anaphylaxis trigger found in skin cleansers, central venous lines, urinary catheters, and other products. Chlorhexidine sensitization was identified in 9.6 % of 228 patients with perioperative anaphylaxis by using specific IgE measurements, BAT, and standardized SPT and intradermal tests [73].

Natural rubber latex allergy remains an important anaphylaxis trigger in many countries. In Israel, prospective use of a 9-item written screening questionnaire before elective Caesarean delivery identified potential clinical reactivity to latex in 14.6 % of 453 women, compared with identification of potential reactivity in only 2.6 % of 460 women who answered a standard verbal inquiry [74].

Of 104 patients with iodinated radiocontrast media (RCM)-induced anaphylaxis, 34.6 % developed it on their first RCM exposure. Patients presenting with shock were older and had more frequent RCM exposures. In the 51 patients who were skin tested, the overall positivity rate was 64.7 % and in those with shock, it was 81.8 % [75].

Although gadolinium-based contrast agents are recommended in patients with a history of RCM-induced anaphylaxis, they, too, can trigger anaphylaxis. Of 614 cases reported to the US Food and Drug Administration’s Adverse Event Reporting System, 43 % were associated with gadopentetate dimeglumine, 29 % with gadobenate dimeglumine, and 17 % with gadoteridol [76].

In a case series of anaphylaxis to IV fluorescein, the most common presentation was hypotension, typically occurring within three minutes of dye infusion [77].

### Diagnosis of anaphylaxis

Diagnosis of anaphylaxis depends on recognition of characteristic symptoms and signs that occur minutes to hours after exposure to a known or potential trigger [1]. High-quality studies relevant to clinical diagnosis and laboratory tests to support the diagnosis of anaphylaxis are being published [78–90] (Table 3).

**Table 3:** **Diagnosis, initial treatment, and self-treatment in community settings**

**Diagnosis [** 78 **–** 90 **]**
In a prospective controlled study of ED patients with anaphylaxis, up-regulation of innate inflammatory gene networks was reported. On ED arrival, two genes were expressed; one hour after arrival, 67 genes were expressed; and three hours after arrival, 2801 genes were expressed. Genomic responses provide new insights into the potential release of a cascade of mediators in anaphylaxis [90].
**Initial treatment [** 91 **–** 103 **]**
Early injection of epinephrine in anaphylaxis, defined as initial injection before ED arrival, significantly reduced the likelihood of hospital admission, compared with initial epinephrine injection after ED arrival [92].
Epinephrine was injected before cardiac arrest in only 23 % of 92 individuals who experienced a fatal anaphylaxis episode [93].
In an observational study, data confirmed the safety of IM epinephrine injection, typically given through an epinephrine auto-injector: (adverse events 1 %, and no overdoses). In contrast, IV bolus injections were associated with significantly more adverse events (10 %) and overdoses (13 %) [99].
In a systematic review and meta-analysis of 27 studies, 4.7% of 4114 patients with anaphylaxis had biphasic episodes (range 0.4% to 23.3%). Patients who presented with hypotension or who had an unknown inciting trigger were at increased risk. The data suggested that for patients with anaphylaxis who are treated successfully in an ED, the duration of observation should be risk-stratified according to the clinical characteristics and severity of the episode [101].
**Long-term management: self-treatment in community settings [** 104 **–** 118 **]**
Patients who were treated in an ED for anaphylaxis benefited from referral to A/I specialists who clarified the diagnosis and correctly identified and confirmed specific anaphylaxis triggers [104].
Novel EAIs are now available in some countries. The compact Auvi-Q can be used correctly on first attempt by 93 % of parents who have never seen or heard it before. The Emerade is available in 0.15 mg, 0.3 mg, and 0.5 mg doses. The 0.3 mg and the 0.5 mg dose EAIs have a 25 mm long needle. EAIs differ significantly with regard to size, ease of carrying, ease of use, needle protection, and robustness. They are not interchangeable [107, 108].

*A/I* allergy/immunology, *ED* emergency department, *EAI* epinephrine auto-injector, *IM* intramuscular, *IV* intravenous

The major anaphylaxis guidelines include the National Institute of Allergy and Infectious Diseases clinical criteria for diagnosis, a practical instrument for rapid identification of patients with a likely diagnosis of anaphylaxis [1, 5, 6, 78]. These criteria are validated for use in EDs and other medical settings and in epidemiological research, in which they are used to define study populations. In order to operationalize them, illustrations [1, 2, 19] and a color-coded chart [78] have been developed.

Under-diagnosis of anaphylaxis is common. Although over 80 % of 7822 responders (most of whom were physicians or allied healthcare professionals) correctly identified anaphylaxis when given a case scenario of a patient with cutaneous involvement, only 55 % recognized anaphylaxis in a case without cutaneous involvement [79]. Importantly, diagnostic confusion has been reported in children hospitalized for life-threatening asthma, some of whom met diagnostic criteria for anaphylaxis [80].

Less common considerations in the differential diagnosis also need to be kept in mind, as in the following examples: Anaphylaxis due to ruptured hydatid cyst can occur in areas *without* endemic *Echinococcus granulosus*, a reminder that untreated patients who have lived in endemic areas remain at life-long risk [81]. Idiopathic systemic capillary leak syndrome has now been documented in children aged 5–11 years; therefore, in the pediatric population, as in adults, it should be considered in the differential diagnosis of anaphylaxis in patients who present with hypotension, shock, and fluid extravasation [82].

In addition, misdiagnosis or over-diagnosis of anaphylaxis can occur, as described in patients with acute multi-system symptoms after ingestion of caustic substances, and those with foreign body inhalation, acute urticaria, or angioedema [83, 84].

### Role of laboratory tests

Serum tryptase levels in blood samples taken 15–180 minutes after symptom onset can support the clinical diagnosis of anaphylaxis in some but not all patients [1, 5, 6]. In food-induced anaphylaxis, single tryptase measurements are typically within the normal reference range; however, in these patients, use of a ratio (peak tryptase level divided by basal level) is reported to improve sensitivity, specificity, and positive and negative likelihood ratios [85].

In perioperative anaphylaxis, serum tryptase levels are not compromised by large volumes of fluid or by resuscitation [86]. An elevated “acute” serum tryptase level (% change >141 %; or absolute tryptase level of >15.7 mcg/L) has been reported to be highly predictive of IgE-mediated perioperative anaphylaxis in a multi-center, retrospective analysis [87].

In post-mortem sera, serum tryptase levels can vary in the same person depending on the site of blood sampling; moreover, elevated levels are not specific for anaphylaxis, but can also occur in people who die from myocardial infarction, asphyxia, or trauma [88].

Even when tryptase levels are within the normal reference range, other mediators of inflammation such as histamine, PAF, PGD2, and LTE4 can be elevated in anaphylaxis [1–6]. Heparin, the contact system, and activation of factor-XII are also involved in anaphylaxis, generating plasma kallikrein that protealyzes kininogen and leads to bradykinin release; moreover, the intensity of contact system activation and bradykinin formation are associated with anaphylaxis severity [89].

Reliance on elevation of only one mediator of inflammation to confirm the clinical diagnosis of anaphylaxis is likely insufficient. In a prospective controlled study of ED patients with moderately severe anaphylaxis, gene expression in peripheral blood samples was profiled in microarrays, differentially expressed genes were identified, and network analysis was used to explore underlying mechanisms [90].

In these patients, on ED arrival, two genes were differentially expressed. One hour post-arrival, 67 genes were expressed; and three hours post-arrival, 2801 genes were expressed. Network analysis demonstrated up-regulation of three inflammatory modules, each containing multiple hub-genes with a central role in the regulation of innate inflammatory responses [90].

Genomic responses in anaphylaxis provide insight into potential release of a cascade of mediators that might play a role in escalation of anaphylaxis symptoms and signs [90].

### Initial treatment of anaphylaxis

Anaphylaxis is a life-threatening medical emergency in which prompt intervention is critical. Principles of treatment remain unchanged [1]; however, recommendations for treatment are based on evidence of increasingly high quality [91–103] (Table 3).

International guidelines concur that epinephrine (adrenaline) is the medication of first choice in anaphylaxis because it is the only medication that reduces hospitalization and death. Its life-saving alpha-1 agonist vasoconstrictor effects prevent and relieve airway edema, hypotension, and shock; its beta-1 agonist chronotropic and inotropic effects increase the rate and force of cardiac contractions, and its beta-2 agonist effects lead to bronchodilation and decreased mediator release [1, 5, 6, 78, 91–93].

Early injection of epinephrine in anaphylaxis, defined as injection before ED arrival, can significantly reduce the likelihood of hospital admission, as compared with initial injection after ED arrival [92]. Delayed injection of epinephrine has been reported in yet another large series of anaphylaxis-related fatalities in which only 23 % of the 92 individuals received it before cardiac arrest [93]. Although epinephrine injection rates remain low in many EDs [94], use of protocols or order sets can significantly improve injection rates [95].

Epinephrine is significantly less likely to be injected in food-induced anaphylaxis than in venom-induced anaphylaxis [38]. Other reasons for failure to inject it include: not giving it because symptoms appear mild or moderate, or there are perceived contraindications to it (pregnancy, older age, or CVD) [95–97], or the patient refuses it [98]. In fact, there are no absolute contraindications to epinephrine [1, 4–6].

More emphasis is needed on prompt recognition and appropriate treatment of anaphylaxis by healthcare professionals. In a 2013 study, although junior doctors’ ability to manage anaphylaxis was somewhat improved compared with a similar study in a 2002 physician cohort, only one-third of them knew the correct dose and route for epinephrine administration [84].

In an ED single-center retrospective observational cohort study of epinephrine safety, 58 % of 573 consecutive patients received epinephrine for anaphylaxis. Only four of 316 patients (1 %) who received an intramuscular (IM) epinephrine injection (most doses administered through an auto-injector) developed adverse events; in contrast, three of 30 patients (10 %) who received IV bolus epinephrine had adverse events. There were no overdoses with IM injection, *versus* an overdose rate of 13 % after IV bolus administration. These data support the safety of the IM route and suggest need for extreme caution when IV bolus doses are administered [99].

International guidelines concur that H_1_-antihistamines, H_2_-antihistamines, and glucocorticoids are second-line [1, 6, 78] or even third-line [5] medications in anaphylaxis. These medications are not life-saving and should not be used as initial or sole treatment. A systematic review of H_2_-antihistamines in anaphylaxis found no randomized controlled trials (RCTs) that met the criteria for inclusion; the H_2_-antihistamines had been studied in acute allergic reactions in which skin symptoms predominated; most (80 %) of the patients had no respiratory or cardiovascular involvement and did not have anaphylaxis [100]. Glucocorticoids are typically administered to prevent biphasic or protracted episodes of anaphylaxis and have little or no effect on initial symptoms and signs [1, 5, 6, 78].

In a systematic review and meta-analysis of 27 observational studies, biphasic episodes occurred in 192 (4.7 %) of 4114 patients with anaphylaxis (range 0.4 % to 23.3 %) [101]. Patients with unknown triggers and those presenting with hypotension were at increased risk; those with food-induced anaphylaxis were at decreased risk. These data suggest that routine prolonged monitoring is unnecessary in patients whose anaphylaxis symptoms have resolved and that the duration of observation should be risk-stratified according to the clinical characteristics and severity of the episode [101].

Methylene blue, a selective nitric oxide cyclic GMP inhibitor, prevents vasodilation and can rapidly reverse the course of anaphylaxis refractory to epinephrine, oxygen, and IV fluid resuscitation. Use in anaphylaxis is based on case reports and extrapolated from use in septic shock. Limitations include adverse events and interference with pulse oximetry [102].

In patients with life-threatening anaphylaxis, cardiovascular collapse, and cardiac arrest, extracorporeal membrane oxygenation (ECMO) is reported to act as a bridge to recovery [103].

### Long-term management of anaphylaxis in community settings: self-treatment

Treatment of anaphylaxis does not end with resolution of an acute episode [1]. Aimed at decreasing anaphylaxis morbidity and mortality in community settings, high-quality studies, including prospective studies of epinephrine auto-injectors (EAIs) and of anaphylaxis education, are being published [104–118] (Table 3). Further studies of the long-term benefits of anaphylaxis education are needed.

Allergy/immunology specialists clarified the diagnosis or accurately identified and confirmed the specific anaphylaxis trigger in 35 % of patients referred from an ED after treatment for anaphylaxis [104]. Visits to an allergist/immunologist reduced the risk of severe anaphylaxis requiring hospital admission [17].

In many countries, EAIs are still unavailable [94]. Where they *are* available, they remain under-prescribed and under-used; as an example, only 11 % of 261 individuals with a history of confirmed anaphylaxis had used an EAI during their most recent episode, and 52 % reported that they had never received an EAI prescription [8, 105]. In another study, only 16 % of 102 patients could demonstrate the correct use of an EpiPen; most patients made at least one mistake and 56 % of them missed three or more steps [106].

New EAIs are being developed and introduced. In the US and Canada, the Auvi-Q is a user-friendly, uniquely-shaped EAI (not a pen), with audio and visual prompts, and needle protection *before* and after use [107]. Most parents (93 %) who had never previously seen an Auvi-Q or a demonstration of its use intuitively used it correctly on their first attempt [108]. In Europe, the Emerade is available in a 0.15, 0.3, and 0.5 mg dose, with a 16 mm needle on the 0.15 mg EAI and a 25 mm (1-inch needle) needle on the 0.3 mg EAI and the 0.5 mg EAI.

EAIs differ significantly with regard to size, ease of carrying, ease of use, needle protection, and robustness. *They are not inter-changeable* [107, 108].

However, manufacturers’ recommendations are similar for all EAIs with regard to storage: keep at 20° to 25 °C (excursions permitted from 15° to 30 °C) to protect the epinephrine solution from degradation and ensure mechanical integrity of the device. In a warm climate, the degradation of epinephrine solution in EAIs is potentially accelerated [109]. If EAIs are subjected to freezing temperatures for a few days, no degradation of epinephrine solution occurs; however, they must be *completely* thawed before use [110].

Long-term follow-up is important to ensure retention of skills by patients, caregivers, and healthcare professionals. As an example,160 medical interns had theoretical training about anaphylaxis and practical demonstrations with EpiPen trainers. They were then randomly assigned into one group tested 3 months later and another group tested 6 months later. Test scores and EAI demonstration skills, including mean time to “inject epinephrine” from EpiPen trainers, were retained at 3 months, but decreased significantly at 6 months [111].

Structured education programs improve anaphylaxis management. In a prospective study, 98 patients with a history of anaphylaxis and 95 caregivers received two anaphylaxis education modules, each lasting three hours. At follow-up 3 months later, those receiving this intervention improved significantly in knowledge and competence in emergency management, and caregiver anxiety was reduced, as compared with controls who received only standard auto-injector training [112].

An anaphylaxis e-learning program for pharmacists developed by the Australian Society of Clinical Immunology and Allergy was compared with lectures or no training at all. Effectiveness was measured using a validated test administered pre-training, immediately post-training, and 3 months and 7 months later. The proportion of e-learners achieving the minimum standard for anaphylaxis knowledge improved from 45 % at pre-test to 87 % at 7 months, confirming long-term improvement *versus* comparators [113].

In many US schools, unassigned EAIs are now available for use in or by students who experience anaphylaxis and do not have a personal EAI available. Efforts to reduce disparities in EAI availability within and between school districts are ongoing [114].

Incorporation of prospectively validated pictograms into a pediatric anaphylaxis emergency action plan potentially increases comprehension of triggers, symptoms and signs, and management [115].

A nurse delivered a training program in person to 4818 individuals at 247 schools and community sites. Written evaluations, online surveys, and phone interviews were used to measure effectiveness. In-person training increased participants’ knowledge and confidence in prevention, recognition, and treatment of anaphylaxis [116].

In some countries, school staff are reported to have a low level of preparedness to prevent and manage food-induced allergic reactions [117]. Guidelines outlining a standardized approach to risk management of patients with food-induced allergic reactions in schools and other community settings have been developed; however, RCTs of the effectiveness of the interventions are needed to strengthen the evidence base for the recommendations [118].

### Long-term management: prevention of recurrence

Regular follow-up of all patients at risk of anaphylaxis is an important principle of long-term risk reduction [1, 5, 6]. High-quality studies, including RCTs that focus on prevention of anaphylaxis are being published [119–150] (Table 4).

**Table 4:** **Long-term management: trigger-specific prevention**

*Food-induced anaphylaxis* [119–134]
Sustained clinical and immunological unresponsiveness to peanut was documented after up to 5 years of peanut oral immunotherapy. Responders had smaller skin test wheals and lower allergen-specific IgE levels at baseline and at the time of post-treatment peanut challenge, compared with non-responders [130].
Sustained clinical and immunological responsiveness to peanut has been documented after up to 3 years of peanut sublingual immunotherapy. Responders had a significant decrease in peanut-specific basophil activation and skin prick test titration with peanut, as compared with non-responders [131].
In selected high-risk infants with eczema, egg allergy, or both, who were aged 4–10 months (inclusive) at study entry, early introduction of peanut snacks (at least 6 grams weekly until age 60 months) prevented development of clinical reactivity to peanut [134].
*Hymenoptera venom-induced anaphylaxis* [135–139]
In 100 adults who completed venom immunotherapy and had live sting challenges to prove efficacy, post-challenge scores on the Vespid Allergy Quality of Life Questionnaire improved significantly, independent of age, sex, or severity of the initial anaphylaxis episode [138].
*Drug/iatrogenic agent-induced anaphylaxis* [140–148]
In a retrospective study of patients with a history of hypersensitivity reactions during anesthesia, comprehensive evaluation, including skin tests and measurement of basal tryptase levels, followed by development of a management plan, minimized risk of subsequent reactions even when a specific trigger such as an antibiotic, neuromuscular blocker, or latex was not identified [145].
*Idiopathic anaphylaxis* [149, 150]
In 20 % of 110 patients with idiopathic anaphylaxis (no identifiable trigger by history, skin tests, or allergen-specific IgE levels), the etiology of the episode was identified by using the ImmunoCAP ISAC 103 allergen array in addition to the ImmunoCAP 250 platform. Omega-5 gliadin and shrimp were the most frequently identified sensitizations among those not previously recognized [149].

### Food-induced anaphylaxis

A detailed history of the anaphylactic episode is a critically important aspect of patient evaluation. In interpreting the history, awareness of emerging new allergens and cross-reactivity of homologous allergenic proteins is helpful [39, 40, 119].

Based on a retrospective study, for predicting outcomes of OFCs, the ratio of food-specific IgE to total IgE was more accurate than specific IgE levels alone in confirming development of tolerance [120].

Component-resolved testing involves identifying specific IgE by using allergenic proteins derived from recombinant DNA technology or purified from natural sources. Among 165 consecutive peanut-sensitized children, those clinically reactive to peanut consistently had significantly higher peanut-specific and Ara h 2-specific IgE antibody levels than those who could tolerate peanut ingestion [121].

Use of the BAT in peanut-sensitized children revealed peanut concentration-dependent up-regulation of CD63 and CD203c in those who were clinically reactive to peanut, contrasting with no significant responses in those who could tolerate peanut ingestion. The BAT reduced the number of OFCs required [122].

The 65–70 % of children destined to outgrow their anaphylactic sensitivity to milk or egg cannot be accurately identified by skin prick tests, nor can specific IgE levels be interpreted as an absolute indication or contraindication to an OFC. However, many caregivers can reliably report that their cow’s milk- or egg-allergic child already eats and tolerates baked goods such as muffins containing small quantities of extensively-heated milk or egg without developing symptoms. For other children, such as a child who has been strictly avoiding cow’s milk and related products, a physician-supervised cow’s milk OFC is strongly recommended [119, 123–125].

Predictors of failure (clinical reactions, including anaphylaxis) during physician-supervised OFCs with baked cow’s milk were: asthma, asthma requiring preventer therapy, IgE-mediated clinical reactions to more than three food groups, and a history of anaphylaxis to cow’s milk [125].

A practical guide to managing cow’s milk allergy and/or egg allergy includes recipes for muffins containing baked milk or baked egg suitable for use in physician-supervised OFCs in hospital settings and instructions for continuing safe introduction of baked milk or baked egg at home after a patient passes an OFC [124]. Useful web-based resources are also available [119].

Adherence, defined as regular dietary consumption of extensively heated cow’s milk or egg, has been documented in two-thirds of children who passed a physician-supervised OFC. Children with asthma appear to be at increased risk of ongoing reactivity after passing an OFC [126].

Based on a validated disease-specific questionnaire, OFCs, regardless of pass or fail outcome, were associated with improved quality-of-life in caregivers of food-allergic children, as compared with caregivers of food-allergic children who did not have OFCs [127].

Liberalizing the diet is important for children who can tolerate baked milk or baked egg because strict elimination diets, particularly those excluding milk, have been associated with linear growth impairment and nutritional deficiencies, exclusion from social activities, and higher direct costs such as medications and indirect costs such as lost wages and household expenses [128, 129].

RCTs confirm that desensitization can be achieved in many patients who are symptomatic after ingestion of highly allergenic foods such as peanut, milk, or egg [130, 131]. During OIT, patients with asthma are at risk for more severe adverse events, including anaphylaxis [20], and are less likely to achieve full desensitization than non-asthmatics [132]. The safety of OIT can be improved by omalizumab pre-treatment and co-treatment [40].

Sustained clinical unresponsiveness to peanut after OIT has been demonstrated in a clinical trial in which children aged 1–16 years were treated with dose increases up to 4000 mg/day peanut protein. Double-blind placebo-controlled OFCs with 5000 mg peanut protein (on average, 12 peanuts) were conducted. Of 39 participants, 24 completed the protocol and 12 passed the OFC 1 month after stopping treatment. This study confirmed sustained unresponsiveness after up to 5 years of peanut OIT. Successful outcomes could be predicted by smaller skin test wheals to peanut and lower peanut-specific IgE levels at baseline and at the time of peanut challenge [130]. In an experimental OIT model, IgG antibodies that act through FcγRIIb to suppress IgE-mediated hypersensitivity have been induced [133].

In a 3 year follow-up study of peanut sublingual immunotherapy, in OFCs performed 2 weeks after treatment ended, 11 % of the 40 patients were able to consume 10 grams of peanut powder, equivalent to 16–18 peanuts. Approximately 98 % of the 18,165 doses were tolerated without severe symptoms beyond the oropharynx. At year 2 of maintenance treatment, responders had a significant decrease in peanut-specific basophil activation and SPT titration compared with non-responders. By study’s end, 10.8 % of 37 patients achieved sustained unresponsiveness to peanut. The drop-out rate was high, mainly due to difficulties in taking the sublingual peanut extract every day [131].

Prevention of clinical reactivity to peanut may eventually be possible. In a landmark RCT, 640 infants with severe eczema, egg allergy, or both, who were age 4–10 months at study entry were assigned to either consume peanuts or avoid peanuts until age 60 months. Randomization was based on pre-existing sensitization to peanut as determined by SPT. The primary outcome was the proportion of children with peanut allergy at age 60 months. In the infants who avoided peanut, the prevalence of peanut allergy was 13.7 %. In those who consumed at least 6 grams of a peanut butter-containing snack per week, it was significantly lower (1.9 %). In these carefully selected high-risk infants, early introduction of peanut prevented subsequent development of clinical reactivity to peanut [134]. Additional studies of prevention strategies are ongoing.

### Stinging insect-induced anaphylaxis

Venom immunotherapy (VIT) effectively reduces susceptible patients’ risk of recurrent SARs. The risk continues to decrease the longer VIT is continued. The currently recommended duration of VIT is 5 years; however, it should be given for more than 5 years to patients who are at particularly high risk for venom anaphylaxis, including the elderly, those with concomitant cardiac and/or pulmonary disease, elevated basal serum tryptase levels, and SARs before or during VIT. Lifelong VIT is the standard of care for patients with clonal mast cell disorders [135]. Omalizumab can be used to facilitate VIT in patients who previously could not tolerate it [136].

Because of the residual risk of SARs after VIT, all patients should carry EAIs indefinitely and continue to avoid *Hymenoptera* stings [135]. When teaching patients to avoid stinging insects and their nests, visual aids should be used to aid in recognition [137].

Sting challenge with a live insect is the only test that accurately evaluates VIT efficacy. In 100 adults who had completed VIT, after their sting challenges, scores on the Vespid Allergy Quality-of-Life Questionnaire improved significantly, independent of sex, age, and severity of the initial anaphylactic episode [138].

In some areas of Australia, jack jumper ant stings are an important cause of anaphylaxis and death. Jack jumper ant VIT effectively prevents anaphylaxis recurrences. Its tolerability and safety are comparable with those of honeybee VIT [139].

### Drug-induced/iatrogenic anaphylaxis

In patients with *a history of anaphylaxis to penicillins*, if at all possible, skin tests, even SPT, and challenge tests should be avoided, because they can trigger anaphylaxis [140, 141].

In contrast, many (90 %) low-risk penicillin allergy-labeled patients *who have no history of anaphylaxis or other penicillin-associated serious life-threatening reactions* can be de-labeled using a risk stratification protocol developed to guide skin testing and challenge tests. Compliance with recommendations for de-labeling can be increased by improved communication with patients and their physicians [142].

In 72 patients without HIV who had a history of sulfonamide adverse drug reactions, the outcomes of trimethoprim-sulfamethoxazole desensitization were evaluated for safety and efficacy after graded administration using short protocols (90 minutes to six hours), one-day protocols, or longer protocols. Desensitization was discontinued due to adverse reactions in only eight patients [143].

Paracetamol, a weak COX inhibitor, rarely triggers symptoms in patients with non-selective hypersensitivity reactions to NSAIDs. Use of a provocation test with paracetamol in children with a history of NSAID allergy is reported to exclude paracetamol as an inducer of anaphylaxis and identify it as a safe treatment option [144].

A retrospective study supports a comprehensive evaluation and management plan for patients with hypersensitivity reactions during anesthesia. Of 72 such patients referred to allergy/immunology, 18 % had positive skin tests to beta-lactam antibiotics, neuromuscular blockers, latex, or other agents. One patient who had an elevated basal tryptase level was diagnosed with mastocytosis. On follow-up, 96 % of the 47 patients requiring subsequent anesthesia tolerated it uneventfully. The two patients with recurrent reactions were later diagnosed with mast cell disorders [145].

IgA-deficient individuals with anti-IgA antibodies can develop anaphylaxis during transfusion of blood or blood products. As compared with time-consuming quantitative assays such as the fluorescein enzyme immunoassay, rapid non-quantitative DiaMed particle gel immunoassays for identifying IgA-deficiency and antibodies to IgA have reasonable sensitivity and specificity. They are potentially useful in emergency situations for diagnosing anti-IgA-related anaphylaxis and identifying donors who can provide IgA-deficient blood products [146].

Use of first-line chemotherapy and monoclonal agents for malignancies and chronic inflammatory diseases prolongs patient survival and minimizes morbidity. Desensitization should be considered when patients develop drug hypersensitivity to first-line agents and no alternative therapies are available, or when alternative treatments are considered to be therapeutically inferior and/or more toxic. The indications, protocols, and outcomes of desensitizations for chemotherapy and monoclonal antibodies in adults and children have been reviewed [147].

Influenza vaccine should not be withheld from egg-sensitized patients, even those with a history of egg-induced anaphylaxis [148]. The egg content of 2015 influenza vaccines is below the threshold needed to trigger reactions in egg-sensitized people. Injected and intranasal influenza vaccines have been studied in 4315 patients with egg allergy, including 656 patients with a history of anaphylaxis to ingested egg. The rare patients with a history of urticaria or anaphylaxis after immunization with influenza vaccine need an allergy/immunology evaluation that includes skin tests to the vaccine itself [148].

### Idiopathic anaphylaxis

In idiopathic anaphylaxis, by definition, meticulous history, skin tests and measurement of allergen-specific IgE levels have not revealed the trigger. In 20 % of 110 patients with previously diagnosed idiopathic anaphylaxis, the etiology of the episode was identified by testing with the ImmunoCAP ISAC 103 allergen array in addition to the ImmunoCAP 250 platform. Omega-5 gliadin and shrimp accounted for 45 % of the previously unrecognized sensitizations. Clinical reassessment substantiated the etiologic link in many patients [149].

In idiopathic anaphylaxis, there are no RCTs of interventions and no standardized treatment recommendations. Rituximab infusions induced remission of idiopathic anaphylaxis in a patient who had anaphylaxis symptoms every 3–30 days despite prophylaxis with high-dose H_1_- and H_2_-antihistamines, a leukotriene antagonist, systemic glucocorticoids, mycophenolate mofetil, omalizumab, and a diet restricted to three foods [150].

## Conclusions

The recommendations for the assessment, management and prevention of anaphylaxis promulgated in the 2011 WAO Anaphylaxis Guidelines are supported and strengthened year-on-year by publication of new, relevant, epidemiological and experimental research findings, including some RCTs.

Important advances in 2014–2015 comprised further elucidation of patient risk factors and co-factors that amplify anaphylaxis, and greater insights into anaphylaxis triggers such as food, venom, and drugs, as well as idiopathic anaphylaxis. A prospective study documented the genomic basis of anaphylaxis and the cascade of mediators released during innate immune system activation in the first three hours after symptom onset.

High-quality observational studies published in 2014–2015 have confirmed that prompt injection of epinephrine reduces hospital admissions and that IM epinephrine injections are 10-fold safer than IV bolus injections. A study of anaphylaxis fatalities found that 23 % of those who died did not receive epinephrine until cardiac arrest. New developments in long-term management of anaphylaxis include novel EAIs, prospective studies of anaphylaxis education, and substantial progress in use of immunomodulation to prevent anaphylactic episodes.

## Abbreviations

ACE: Angiotensin-converting enzyme
Alpha-gal: Galactose-alpha 1,3-galactose
ASA: Acetylsalicylic acid
BAT: Basophil activation test
CVD: Cardiovascular disease
EAI: Epinephrine auto-injector
ECMO: Extracorporeal membrane oxygenation
ED: Emergency department
ICD: International Classification of Diseases
ICON: Anaphylaxis: International Consensus on Anaphylaxis
IM: Intramuscular
ISM: indolent systemic mastocytosis
IV: Intravenous
MCAS: Mast cell activation syndrome
NMBA: Neuromuscular blocking agent
NSAIDs: Non-steroidal anti-inflammatory drugs
OFC: Oral food challenge
OIT: Oral immunotherapy
PAF: Platelet-activating factor
RCM: Radiocontrast media
RCT: Randomized controlled trial
SAR: Systemic allergic reaction
SPT: Skin prick tests
VIT: Venom immunotherapy
WAO: World Allergy Organization
